# Antitumor activity of genetically engineered NK-cells in non-hematological solid tumor: a comprehensive review

**DOI:** 10.3389/fimmu.2024.1390498

**Published:** 2024-04-17

**Authors:** Chinmayee Priyadarsini Dash, Dhruba Sonowal, Prachi Dhaka, Rohit Yadav, Dewan Chettri, Bibhu Prasad Satapathy, Pooja Sheoran, Vivek Uttam, Manju Jain, Aklank Jain

**Affiliations:** ^1^ Non-Coding Ribonucleic Acid (RNA) and Cancer Biology Laboratory, Department of Zoology, Central University of Punjab, Bathinda, Punjab, India; ^2^ Department of Biochemistry, Central University of Punjab, Bathinda, Punjab, India

**Keywords:** CAR-NK cell therapy, solid tumor, non-hematological tumor, cancer immunotherapy, Genetically engineered CAR-NK cell

## Abstract

Recent advancements in genetic engineering have made it possible to modify Natural Killer (NK) cells to enhance their ability to fight against various cancers, including solid tumors. This comprehensive overview discusses the current status of genetically engineered chimeric antigen receptor NK-cell therapies and their potential for treating solid tumors. We explore the inherent characteristics of NK cells and their role in immune regulation and tumor surveillance. Moreover, we examine the strategies used to genetically engineer NK cells in terms of efficacy, safety profile, and potential clinical applications. Our investigation suggests CAR-NK cells can effectively target and regress non-hematological malignancies, demonstrating enhanced antitumor efficacy. This implies excellent promise for treating tumors using genetically modified NK cells. Notably, NK cells exhibit low graft versus host disease (GvHD) potential and rarely induce significant toxicities, making them an ideal platform for CAR engineering. The adoptive transfer of allogeneic NK cells into patients further emphasizes the versatility of NK cells for various applications. We also address challenges and limitations associated with the clinical translation of genetically engineered NK-cell therapies, such as off-target effects, immune escape mechanisms, and manufacturing scalability. We provide strategies to overcome these obstacles through combination therapies and delivery optimization. Overall, we believe this review contributes to advancing NK-cell-based immunotherapy as a promising approach for cancer treatment by elucidating the underlying mechanisms, evaluating preclinical and clinical evidence, and addressing remaining challenges.

## 1 Introduction

Despite recent breakthroughs in cancer diagnosis and treatment technologies, cancer continues to rank as the second most common cause of death globally (1). Among all cancer types, non-hematological or solid cancers are widespread (89.4%) and trickier to handle compared to blood-related ones, i.e., hematological (10.6%) (2). Wrapping our heads around these non-hematological tumors like breast, lung, colorectal, ovarian, and liver cancers and finding effective treatments resembles tackling a more challenging puzzle. The treatment approach for solid cancers is highly diverse such as surgery, chemotherapy, radiation therapy, targeted therapy, and hormone therapy depending on the type and stage. While several effective medications, including Tamoxifen (3), Bevacizumab (4), Erlotinib, and Cetuximab (5), have been developed, the effectiveness of these drugs varies among different cancer patients and types (6). These medications and treatment modalities have some limitations or offer little advantages in specific circumstances, primarily due to the focus of conventional treatments on the tumor itself (7). Moreover, cancer cells can sometimes evade these conventional treatments.

Immunotherapy is emerging as a prominent treatment strategy over traditional therapies as it utilizes the body’s immune system to recognize and target cancer cells. This approach is crucial because of its precision targeting, and it can be combined with other treatments to create synergistic effects, thus amplifying the overall therapeutic impact. Immunotherapy represents a paradigm shift by offering a focused, flexible, and potentially more effective method of treating cancer. For example, chimeric antigen receptor T cells (CAR-T), anti-PD-1/PD-L1 therapies and other checkpoint inhibitor-based therapies have revolutionized cancer treatment and shown remarkable success in some cases (8). However, it is also true that these therapies are not universally effective for all patients or all types of cancer.

Importantly, NK cells carry a lower risk of inducing graft-versus-host disease than CAR-T cell-based therapies (9, 10). Additionally, NK-cell therapy offers the advantage of being derived from healthy donors (allogeneic therapy), presenting an “off-the-shelf” treatment option, in contrast to the challenges associated with autologous CAR-T-cell therapies and subsequent modification for reinfusion (11, 12). This becomes challenging, especially for individuals with cancer who have undergone extensive pretreatment and cannot contribute a sufficient number of normal T-cells, posing a hurdle to CAR-T cell production (13). Moreover, CAR-T cells are associated with surplus side effects such as neurotoxicity (13), and their generation involves a time-consuming and expensive process (14). In contrast, the safety profile of NK cell therapy is further enhanced by a reduced likelihood of cytokine release syndrome (CRS), a potentially severe side effect associated with various immunotherapies, including CAR-T cell therapy (15, 16).

This review article will provide a comprehensive discussion on utilizing CAR-NK cells for treating cancer patients, focusing on solid tumors and exploring prospective applications in cancer immunotherapies.

To conduct our literature review, we utilized reputable databases such as Google Scholar and PubMed. We conducted search using string "CAR-NK cell AND Immunotherapy in cancer NOT review" without imposing any time constraints. Subsequently, we performed scientific screening, excluding papers related to hematological solid tumors. Following this, we carefully scrutinized and included studies involving human cell lines, as well as studies employing both human cell lines and mouse models. This meticulous approach ensures that the literature synthesized in our review is both substantial and directly pertinent to our topic.

## 2 Chimeric antigen receptor-NK cells

CAR-NK cell is a type of immune cell engineered to express CAR, combining the specificity of an antibody with the potent cytotoxicity of natural killer (NK) cells (17). CAR-NK cells are designed to target and destroy cancer cells more effectively, offering a potential immunotherapy approach for certain types of cancer. Unlike CAR-T cells, which use T cells as the base cell type, CAR-NK cells utilize natural killer cells, which have inherent tumor-killing abilities and may offer advantages such as safety and reduced risk of graft-versus-host disease (18, 19).

### 2.1 Structure of CAR-NK cells and genesis

Recent advances in research have focused on genetically modifying NK cells to enhance their effectiveness in targeting and killing cancer cells, thereby generating targeted effector cells (20). One approach to boosting killing efficacy involves engineering NK cells to express a CAR enabling CAR-NK cells to specifically target tumor cells expressing a particular antigen (20, 21).

The functional structure of CAR-NK cells consists of 3 major domains: an extracellular domain, a transmembrane domain, and an intracellular signaling domain (22–24). The extracellular domain outside the cell membrane contains a signal peptide, an antigen recognition region, and a spacer. This region houses a single-chain variable fragment (scFv) derived from monoclonal antibody that recognizes tumor-specific antigens (20, 25, 26). The linker connects the variable sections of the heavy and light chains of the antibody molecule (23), influencing the extent to which a CAR can effectively identify the target epitope. The hinge, or spacer, connects the scFv to the transmembrane domain, affecting CAR bearing immune cells specificity. Modifying the length of the spacer could potentially enhance the alignment between CAR-bearing cells and target cells, facilitating the formation of an optimal immunologic synapse (IS) and enhancing specificity (21). The transmembrane domain, responsible for conveying signals from the extracellular domain to the intracellular domain, binds the CAR into the NK cell membrane (20). Commonly used transmembrane components for CAR-NK cells are modified from CD3ζ, CD8, and CD28, with others like NKG2D, 2B4, and DNAM1 also under investigation. The intracellular signaling domain primarily comprises the CD3ζ chain of the T-cell receptor (TCR) as a signaling domain, along with costimulatory molecules (20, 27, 28). Different generations of CARs result from changes to the intracellular signal transduction domain (29).

**Figure 1:**
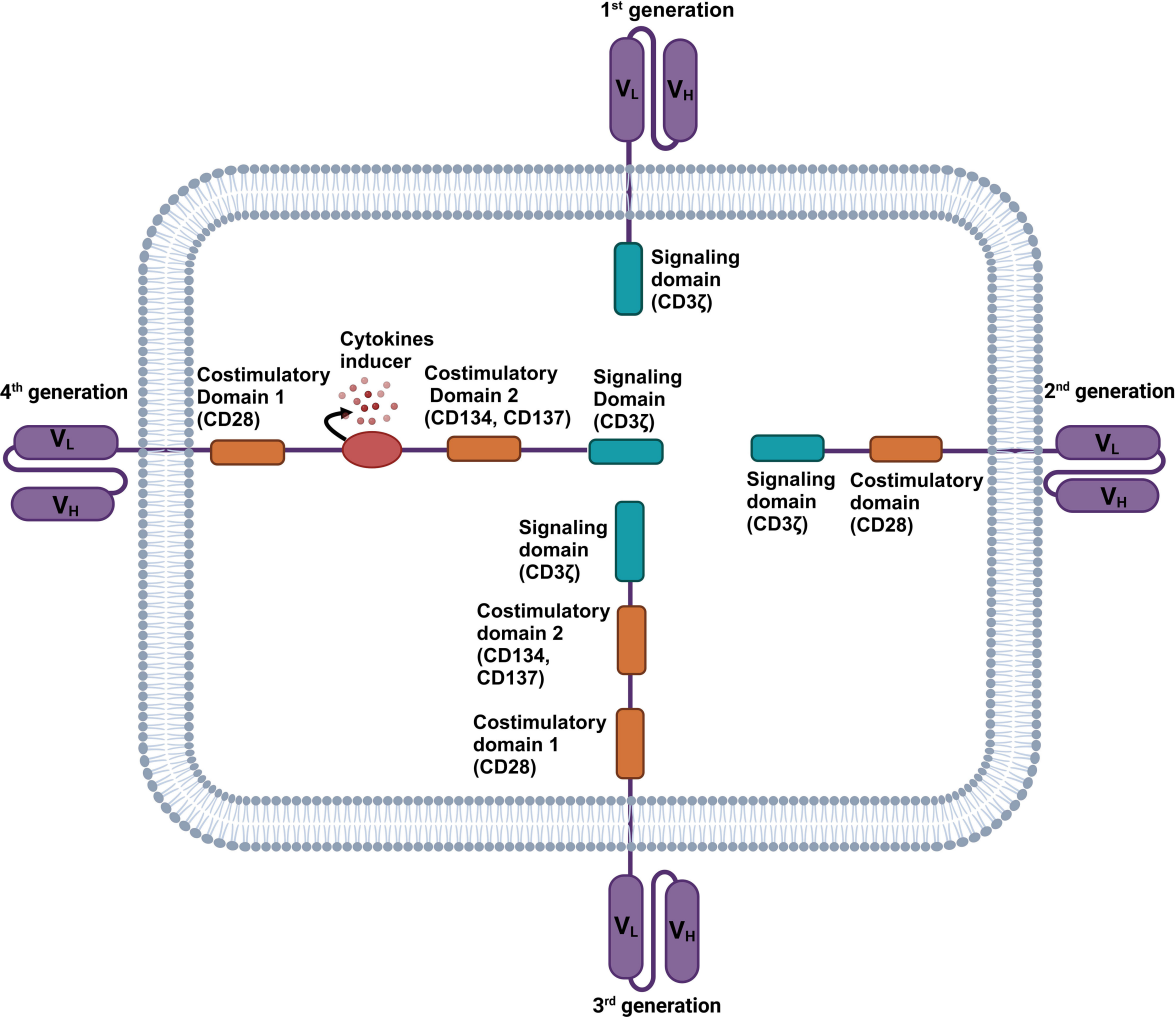
Structure of four generations of chimeric antigen receptor (CAR) NK cells. The first generation of CAR-NK cell consists of a CD3ζ signaling domain. The second generation CAR-NK cell consists of a signaling domain (CD3ζ) and a costimulatory domain (CD28). Third generation CAR-NK cells consist of a signaling domain (CD3ζ) and two costimulatory domains (CD28 and CD137 or CD134). Fourth generation CAR-NK cells consist of a signaling domain (CD3ζ), multiple costimulatory domains (CD28 and CD137 or CD134), and a cytokine inducer domain.

On the basis of intracellular signalling domains, four generations of chimeric antigen receptor NK cells have been developed till now as illustrated in **Figure 1**. The first generation of CAR-NK cells included a scFv domain to detect tumor antigens and an intracellular CD3ζ signaling domain (27). The second generation was prepared with the addition of costimulatory domain CD28 that improved cell multiplication and cytotoxicity. Third generation consists of multiple signaling and costimulatory domains like CD134 (OX40), CD28, and CD137 (4-1BB) to improve the CAR-NK cell (20, 29). CARs in the fourth generation are engineered to produce cytokines. They are frequently equipped with multiple costimulatory molecules like CD134, CD28, or CD137, to boost antitumor potential by rousing the innate immune system (18, 30).

### 2.2 Mechanistic insights of CAR-NK cell

NK cells are cytotoxic lymphocytes derived from common lymphoid progenitor (CLP) cells in the bone marrow and secondary lymphoid tissues (31). They are capable of identifying and eliminating malignant and virally-infected cells. Although these are derived from CLP, they play a primary role in the innate immune system by working against viruses, microbial pathogens, parasites, and tumor immunosurveillance (32). Morphologically, these are big granular lymphocytes identified particularly by the expression of CD56 and CD16 biomarkers (33).

NK cells contain two different kinds of receptors: (a) inhibitory receptors, which bind to MHC class 1 molecules and block NK cells from killing normal cells (34); (b) activating receptors that induce activation signals and trigger NK cell cytotoxicity. They recognize their targets either by missing self-mechanism in which NK cells perceive the presence of normal MHC-I proteins on a cell or by the balance signal mechanism, where the balance between signals from activating and inhibitory receptors is determined (35). Although NK cells do not express antigen-specific receptors, they can identify transplanted tissue that expresses MHC-I, either haploidentical or allogeneic (36).

NK cells are normally activated by the interleukins (IL) produced by activated macrophages and dendritic cells (DCs) such as IL-15, IL-12, IL-18, IL-35, IFN-α, IFN-β, IL-27, IL-1β, and IL-23. These activated NK cells release granzyme, perforin, effector molecules of the TNF family, and FAS- ligand to induce apoptosis and necrosis of the target cells. These cytotoxic contents are released upon target cell identification, triggering secretory lysosome exocytosis. The granzymes enter the target cell’s cytoplasm with the assistance of perforin, where they cleave several targets, including caspases, which lead to cell death (37, 38). They sometimes show antibody-dependent cell cytotoxicity (ADCC) (39), which senses non-specific alterations caused by cellular stress, infections, or malignant transformations (40).

**Figure 2:**
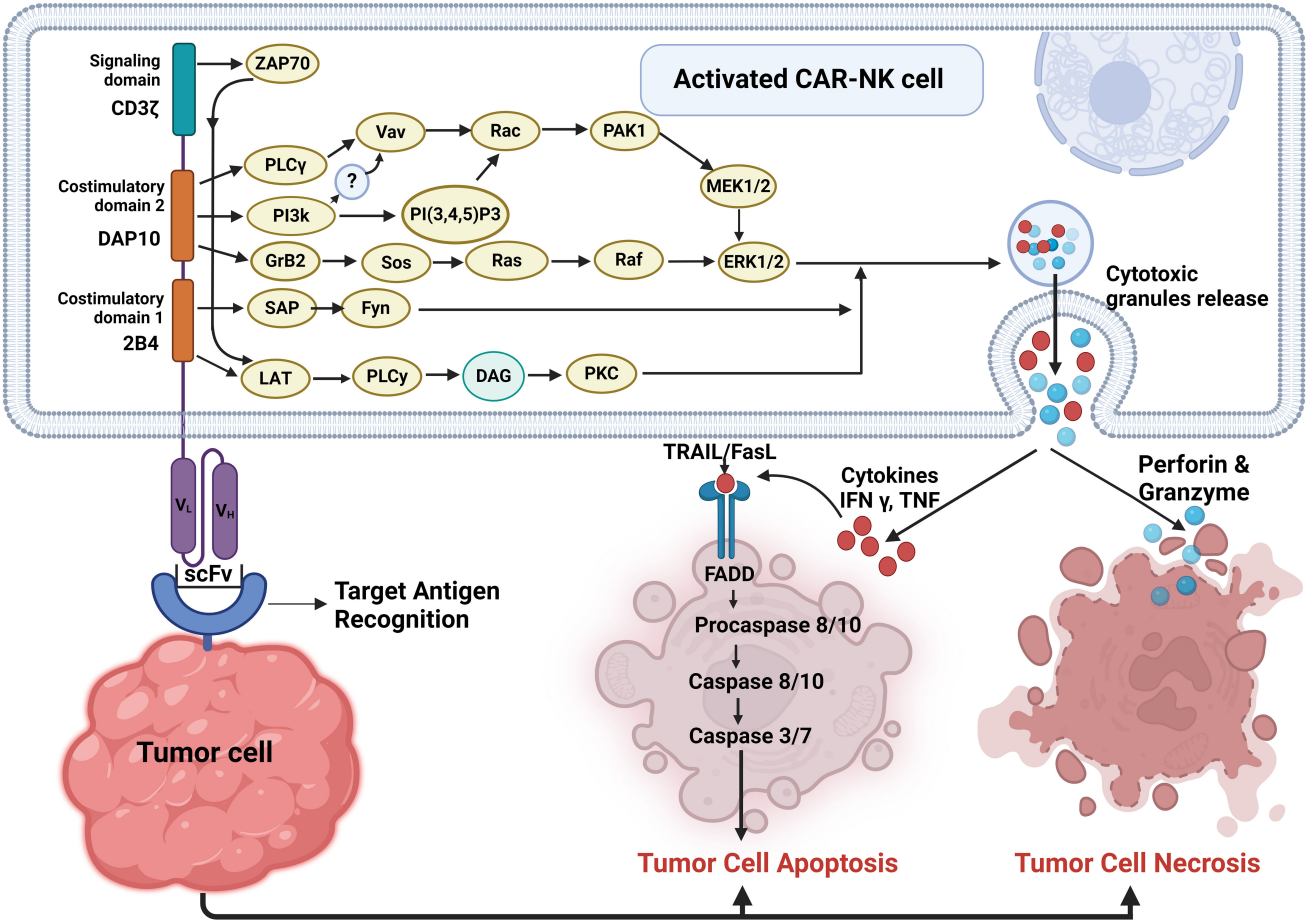
Pathways involved in activated CAR-NK cell-mediated tumor cell death. Upon target cell recognition, CAR-NK cells get activated and can induce tumor cell death through two main pathways: apoptosis and necrosis.

CAR-NK cells exhibit their anticancer activity by inducing both necrosis and apoptosis in tumor cells. These are capable of recognizing and targeting tumor cells expressing specific antigens through their chimeric antigen receptors (CARs). Upon engagement with these target cells, CAR-NK cells can trigger both necrotic and apoptotic pathways within the tumor cells, resulting in their destruction (41) as shown in **Figure 2**. This dual mechanism of action enables CAR NK cells to effectively eliminate cancer cells while minimizing the potential for immune evasion and resistance mechanisms employed by tumors. Thus, harnessing the cytotoxic potential of CAR-NK cells holds significant promise for the development of novel and targeted immunotherapies against cancer.

## 3 CAR-NK and non-hematological solid tumors

**Figure 3:**
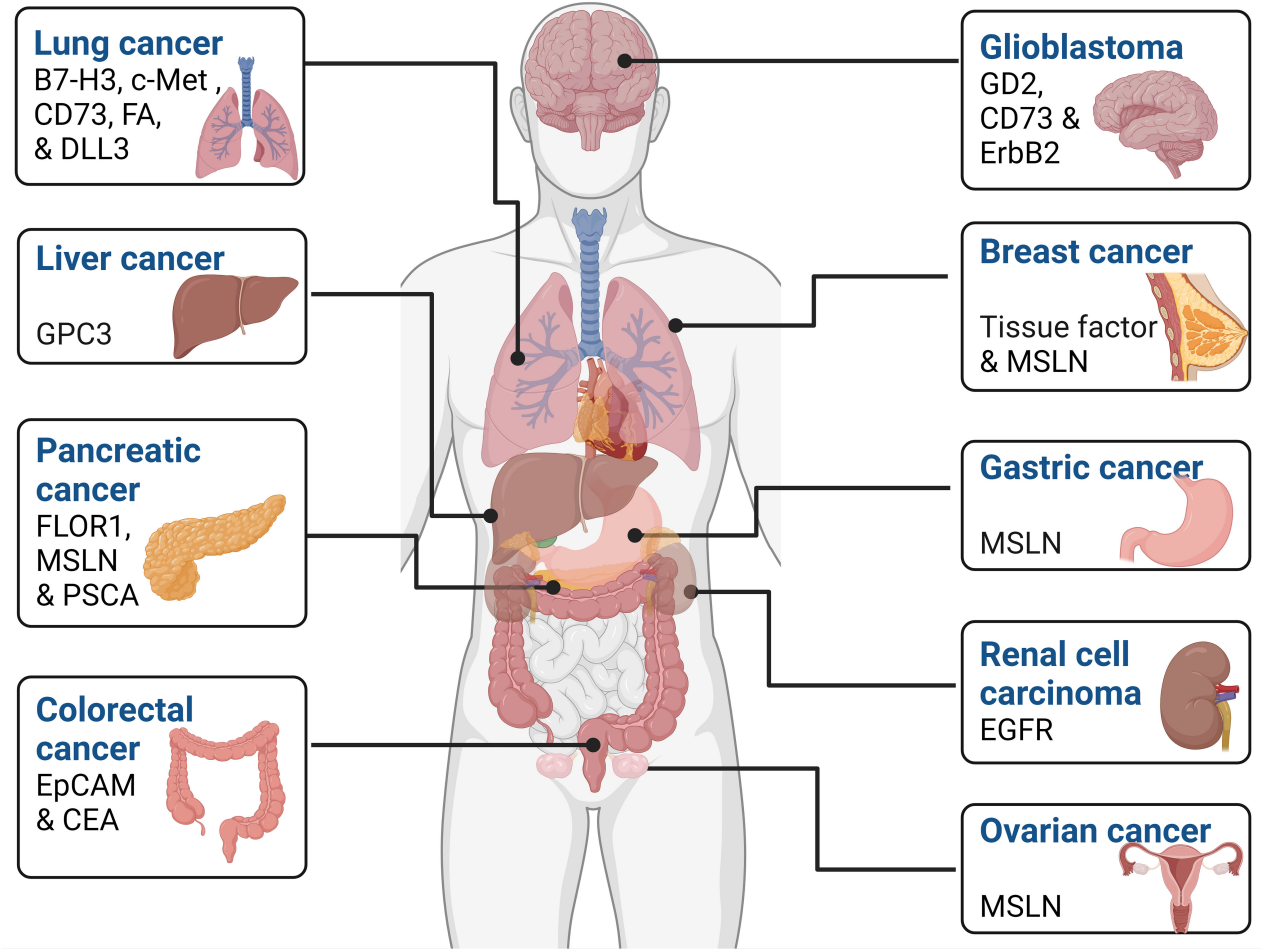
Tumor-associated antigens expressed by various cancers. These markers can be used as a potential therapeutic target in various cell-based cancer immunotherapy such as CAR-NK cell therapy.

A substantial body of research, encompassing both *in vivo* and *in vitro* studies, has extensively examined the effectiveness of CAR-NK cells against a diverse range of solid tumors. These studies have covered glioblastoma, breast, ovarian, lung, liver, colorectal, ovarian, and pancreatic cancers. The wealth of preclinical data derived from these investigations has laid the foundation for the initial clinical trials employing CAR-NK cells in treating solid malignancies. The subsequent sections offer a thorough examination of how engineered CAR-NK cells can finely tune their functionality across various types of cancers by targeting tumor-associated antigens which display differential expression in several malignancies are depicted in **Figure 3**.

### 3.1 Ovarian cancer

The primary challenge for any cancer immunotherapy is choosing a specific marker(s) or target that can differentiate the tumor tissue from healthy tissue. Surprisingly, recent research found mesothelin (MSLN), a glycosylphosphatidyl inositol-linked membrane glycoprotein, is present at significantly lower levels in mesothelial cells of the pleura, peritoneum, and pericardium of healthy persons (42, 43) than several cancer types, including ovarian cancer. Hence, nowadays, MSLN is a hot topic of research and the target for immunotherapy work for CAR-NK based immunotherapies (44).

In this regard, Cao et al. (45) first assessed the MSLN expression in 66 primary ovarian cancer patients and 24 normal ovarian samples. They found that just one normal case, 1/24 (4.2%), had a mild expression, compared to 84.9% (56/66) of the ovarian cancer samples showing strong expression of MSLN. Later, with the help of western blot analysis, the authors found that the MSLN protein expresses higher in OVCAR-3 and SK-OV-3 human ovarian cancer cell lines and is almost negligible in the control human liver SKHEP-1 cancer cell line. These findings show that MSLN overexpresses in ovarian cancer cell lines (45).

To demonstrate the mechanistic effect of CAR-NK cells in ovarian cancer, the authors have generated MSLN- or CD19-CAR molecule by cloning the genes of anti-MSLN scFv, and anti-CD19 scFv, along with CD8 leader, CD8 hinge, CD28 transmembrane, and composite CD28-CD137-CD3ζ intracellular signaling domains into the lentiviral vector pWPXLd-2A-eGFP through PmeI and SpeI cloning sites. MSLN- or CD19-specific CAR vectors were transduced into NK-92 cells to produce MSLN- or CD19-CAR NK cells. Interestingly, it was observed that coculture with OVCAR-3 GL and SK-OV-3 GL cell lines at high (4:1) E: T (effector: target), notably, MSLN-CAR NK cells exerted higher cytotoxicity than either CD19-CAR NK or parental NK-92 cells. However, the cytotoxicity efficacy of MSLN-CAR NK, CD19-CAR NK, and parental NK-92 cells was nearly unchanged for MSLN-negative cells (SK-HEP-1 GL) (45).

Furthermore, moving from *in vitro* studies to *in vivo* efficacy, the authors established subcutaneous and intraperitoneal tumor models in six-to eight-week-old female NSG mice (45). On the first day, SK-OV-3 cells were injected subcutaneously into the animals, and on the fifteenth day, MSLN- or CD19-CAR NK cells were administered intravenously. Tumor volume grew significantly in the negative control (NC) and CD19-CAR NK groups compared to the baseline 5 cm, but it remained relatively stable in the MSLN-CAR NK group. Because of this, the MSLN-CAR NK group’s assessed average tumor weight (0.12 g) was considerably lower than the NC (0.72 g) and CD19-CAR NK (0.53 g) groups’ respective average tumor weights. Based on the subcutaneous model, these finding indicate that MSLN-CAR NK cells have a high potential for curing ovarian cancer (45). They found that CAR-NK92 cells guided by mesothelin (MSLN) effectively eliminate MSLN expressing cell lines (OVCAR-3 and SKOV3) as well as subcutaneous and intraperitoneal ovarian cancer in xenograft models made with NSG (NOD scid gamma) mice. They further observed that this treatment also increases the mice’s longevity (45).

OVCAR-3 GL cells were delivered intraperitoneally to create intraperitoneal ovarian cancer models on day zero. When the mice were exposed to bioluminescence imaging on day 15, tumor cells had significant intraperitoneal growth. After this, these mice were split into NC, MSLN-CAR NK, and CD19-CAR NK. That same day, the mice were given intraperitoneal effector cells or PBS. MSLN-CAR NK cells induced significant tumor regression, whereas tumors with NC and CD19- CAR NK groupings continued to show progression. After receiving PBS or CD19-CAR NK cell injections, mice in the NC or CD19-CAR NK groups displayed a notable improvement in bioluminescence signals in their abdominal cavity on days 22 and 36 as compared to day 15, with the NC group exhibiting the most significant increase. On days 22 and 36, animals that received injections of MSLN-CAR NK cells showed a substantial decrease in intraperitoneal bioluminescence signals (45).

Furthermore, mice in the MSLN-CAR NK group survived significantly longer than those in the NC and CD19-CAR NK groups (45). Thus, the collective findings of these investigations indicate that MSLN-CAR NK cells may be effectively employed to eliminate tumor cells, delay ovarian cancer development *in vivo* and *in vitro*, and extend the survival of tumor bearing mice.

### 3.2 Gastric cancer

According to the 2020 GLOBOCAN data, gastric cancer accounted for approximately 768,793 deaths globally, making it the fourth leading cause of cancer-related mortality (2). Despite a gradual decline in both incidence and mortality rates due to improved access to various treatment modalities, the five-year survival rate for advanced GC remains poor worldwide (46, 47). Therefore, it is crucial and imperative to undertake investigations and explore new innovative treatment approaches for GC.

Given the gravity of gastric cancer, Cao et al. (48) developed MSLN-specific CAR NK-92 (MSLN-CAR NK) cells and assessed their antitumor effects using a gastric cancer mouse model (48). Initially, they identified MSLN expression in 75 primary gastric cancer tissue samples and 20 normal gastric samples. Immunohistochemical staining revealed strong MSLN expression in 54.7% (41/75) of GC tissue samples compared to normal gastric tissues. Subsequently, the authors used immunoblotting analysis to assess MSLN expression in N87, MKN-28, and AGS GC cell lines and Huh-7 liver cell lines. MSLN expression was almost negligible in Huh-7 liver cell lines compared to gastric N87, MKN-28, and AGS cell lines, indicating elevated MSLN expression in primary samples and GC cell lines.

Furthermore, the authors designed MSLN- or CD19-CAR NK molecules containing intracellular domains of co-stimulatory 2B4 and CD3ζ, a CD8 hinge, an NKG2D transmembrane region, and MSLN or CD19 scFv. MSLN- or CD19-CAR NK cells were created by transducing NK-92 cells with the respective CAR vectors. To assess the cytotoxicity of CAR-NK cells against GC cell lines *in vitro*, the authors conducted a 6-hour killing experiment using MSLN- and CD19-CAR NK cells for GL-expressing cells (N87 GL, MKN-28 GL, AGS GL, and Huh-7 GL). The results demonstrated that MSLN-CAR NK cells were more cytotoxic than parental NK-92 or CD19-CAR NK cells. Conversely, for Huh-7 GL, all three cell types exhibited minimal cytotoxicity efficacy, suggesting intrinsic target-dependent cytotoxic activity of MSLN-CAR NK cells *in vitro* (48).

Additionally, to evaluate *in vivo* efficacy, the authors created a subcutaneous model by inoculating N87 cells into six- to eight-week-old female NSG mice on day 0 and treating them intravenously with MSLN- or CD19-CAR NK cells on days 8, 15, and 22. They observed that while tumor volume in the NC and CD19-CAR NK groups significantly increased over the baseline (50 mm3), the calculated tumor volumes in the MSLN-CAR NK group remained relatively stable and modest. The MSLN-CAR NK group exhibited a measured average tumor weight of 0.23 g, considerably less than that of the NC (1.22 g) and CD19-CAR NK (1.06 g) groups. Importantly, mice given CAR-NK cell treatment showed no overt signs of harm to vital organs. These findings suggest that MSLN-CAR NK cells may have a high potential for eradicating subcutaneous GC (48).

Additionally, the authors established an intraperitoneal GC model by injecting MKN-28 GL cells into the peritoneal cavity of mice on day 0. BLI on day 10 revealed a noticeable increase in tumor cells inside their bodies. However, tumors in the NC and CD19-CAR NK groups continued to grow, as seen by BLI on days 15 and 30, while MSLN-CAR NK cells showed a significant reduction in the growth of MKN-28 GL gastric cancer cells. Moreover, the MSLN-CAR NK group of mice lived noticeably longer than the NC and CD19-CAR NK groups.

These findings indicate that MSLN-CAR NK cells may successfully eliminate intraperitoneal GC cells and increase the tumor-bearing mice’s survival duration.

### 3.3 Breast cancer

BC ranks as the most prevalent cancer among women (49) and is the fifth leading cause of cancer-related deaths globally (2). Breast cancer is typically classified into three categories determined by molecular and histological findings. These categories consist of breast cancer characterized by the presence of estrogen and progesterone hormone receptors (ER+ or PR+), breast cancer with amplified human epidermal growth factor receptor 2 (HER2+), and triple-negative breast cancer (TNBC), which does not exhibit estrogen receptor (ER-), progesterone receptor (PR-), or human epidermal growth factor receptor 2 (HER2-) expression (50–53). Among these, BC-expressing hormone receptors (ER^+^ or PR^+^) account for 60-70% of BC cases in developed nations, occuring predominantly in premenopausal women (53). Breast cancers that are HER2-enriched account for 10-15% of all cases. It is distinguished by strong HER2 expression in the absence of ER and PR (54–57). However, TNBC accounts for ~15% of all breast cancers diagnosed worldwide and is the most challenging in comparison to other groups of BC (53, 55, 58). Some studies suggested that surface molecules such as mesothelin (MSLN) and tissue factor (TF) are highly expressed in TNBC cancer cells and can also be a potential targeted surface molecule for TNBC therapy (49).

In a study conducted by Yang et al. (2023) taking Mesothelin (MSLN) as their target in triple-negative breast cancer (TNBC), they developed MSLN CAR-NK cells from induced pluripotent stem cells (iPSCs) whose efficiency was tested in several preclinical models (49). Roughly 67% of TNBC instances demonstrate significantly elevated MSLN levels (59).

The authors tested 39 women with breast cancer, of which only 2 were diagnosed with TNBC. MSLN was shown to be present in both TNBC tumor samples (49). They next induced pluripotent stem cells (iPSCs) that express mesothelin (MSLN-iPSC). The cells were then developed into CAR-NK cells that aim to target mesothelin (MSLN-NK) (49). The efficiency of MSLN-NK cells was then investigated in several preclinical models (49).

MSLN-NK cells demonstrated significant cytotoxicity against MDA-MB-231 (MD231) cells *in vitro* and decreased tumor volumes in a TNBC-CDX (cell line-derived xenograft) mouse model. They eradicated patient-derived primary cancer stem cells (PSPCs) and patient-specific organoids (PSOs) obtained from TNBC tumor samples, indicating that MSLN-NK may be a promising therapeutic option for TNBC patients (49). They employed the TNBC cell line MD231 to assess the efficacy of MSLN-NK cells. The MSLN-NK cells almost wholly eradicate the MD231 target cells. Flow cytometry revealed that MDA-MB-231 cells displayed positive mesothelin expression (49).

To assess the *in-vivo* efficacy, the B-NDG (Biocytogen pharmaceuticals) mouse model was selected to establish the TNBC-CDX model by transplanting the MD231 cell line. After the xenografts produced solid tumors, one mouse was killed, and the tumor tissue was taken for IHC analysis (49). The IHC analysis confirmed positive expression of MSLN in the tumors of the TNBC-CDX model. The TNBC-CDX model mice were given MSLN-NK cells, iPS-NK cells, or saline via weekly injections beginning on day 0. On day 48, the tumors were assessed for volume and then excised when the mice were sacrificed. It was observed that the MSLN-NK group had decreased wet weights and volumes of tumors compared to the control iPS-NK and saline groups. Notably, the mice’s weights remained largely steady throughout the experiment, showing that MSLN-NK cell treatment had no adverse effects on their health (49). Thus, MSLN-NK cells derived from iPSCs can effectively kill MSLN-positive TNBC tumor cells *in vitro* and *in vivo* (49).

Furthermore, for addressing a target surface molecule for TNBC therapy, Hu (55) found that Tissue Factor (TF) serves as a potential target for cancer stem cells (CSCs) that can be derived from various sources such as human breast, lung, and ovarian cancer cell lines. This includes triple-negative breast cancer (TNBC), tumor xenografts, as well as breast cancer patients (55, 60). TF is a commonly named coagulation factor III and CD142. It is a forty-seven kilodalton cell surface receptor attached to the membrane (61–63). TF is also found to be highly expressed in many solid cancers (60, 64–66) and is not expressed in normal tissues and organs (67–69). Thus, the author determined TF as a helpful surface target in patients with TNBC (55).

Hu (55) created the TF-targeting immunotherapeutic agent called ICON to target TF for immunotherapy (70–72). ICON is an immunoconjugate resembling a chimeric antibody, existing as a homodimer with a molecular weight of 210 kilodaltons (kDa) that comprises either murine or human fVII full-length peptide composed of 406 amino acid residues, which is linked to the Fc region of IgG1 (70–73). The author’s laboratory enhanced ICON to create a second-generation version known as L-ICON or L-ICON1 by eliminating the heavy chain responsible for fVII procoagulant activity (60). Natural killer (NK) cells play a pivotal role as CD16+ ADCC effector cells employing ICON, L-ICON in the effectiveness of antibody immunotherapy (60, 74). Thus, for an effective approach to TNBC treatment and to resolve NK cell dysfunction along with improving the effectiveness of L-ICON, the author created TF-targeting CAR-NK cells by IL-2 independent human NK cell line known as NK92MI (55).

In this investigation, TF-targeting CARs were constructed using a Kozak sequence and a human fVII light chain serving as the TF-targeting region. This TF targeting region is attached to the CD28 transmembrane and cytoplasmic domains with or without a hinge region derived from human IgG1 and then by 4-1BB and CD3ζ human cytoplasmic domains. TF-targeting CARs were called TF-CAR1 monomers and dimers, containing a hinge region (55).

Accordingly, the author introduced full-length human CD16 (fCD16) into NK92MI cells via transfection using the pcDNA3.1(+) plasmid, resulting in the creation of a NK92MI/fCD16 that is capable of effectively facilitating ICON, L-ICON, and ADCC. The NK92MI/fCD16 transduced by lenti-CAR1 dimer and monomer viruses. These NK92MI/fCD16 cells stably transduced by lentivirus were named NK-CAR1 dimer and monomer (55).

The cytotoxicity of NK-CAR1 dimer and monomer to kill human TNBC cells (MDA-MB-231) was then examined. The TF-CAR-NK cells demonstrated notable direct cytotoxic effects on MDA-MB-231 cells using both CAR1 monomer and dimer, indicating that TF-CAR-NK cells possess the capability to eliminate TF-positive TNBC cells (55).

After examining NK-CAR1 dimer and monomer cytotoxicity, their capacity to facilitate L-ICON ADCC against both human and murine TNBC cells was determined. He observed that TF-CAR-NK monomer and dimer cells demonstrated increased cytotoxicity against MDA-MB-231 in the presence of L-ICON1 compared to those without L-ICON. They also examined that in the absence of NK cells or complement, ICON alone failed to arrest the *in vitro* proliferation of TF-positive cancer cells. These findings indicate that TF-CAR-NK cells have the potential to function as effector cells in mediating L-ICON ADCC, resulting in a more pronounced effect compared to TF-CAR-NK cells acting alone (55).

They then proceed towards the *in vivo* study by assessing the safety and the therapeutic effectiveness in an orthotopic TNBC CDX NSG mouse model. Orthotopic mice models of tumor line-derived xenografts were generated by injecting female NSG aged 4-6 weeks with 5×10^5^ human TNBC MDA-MB-231/Luc+GFP cells in 50 µl of PBS into mammary gland. After treatment with 2×10^6^ NK-CAR1 monomer or dimer cells tumor growth had been markedly reduced. NK-CAR1 dimer cells showed a marginal improvement in efficacy compared to NK-CAR1 monomers for treating TNBC CDX as demonstrated by reduced tumor volume and weight compared to control tumor (55).

The efficacy and safety of TF-CAR1 dimer treatment were then demonstrated in NSG mice using the orthotopic TNBC PDX model. An orthotopic TNBC PDX (patient-derived xenograft) model was created using a TNBC PDX donor NSG mouse (NOD SCID gamma, lacking mature B cells, T cells, NK cells, and absent complement) with a BRCA1 mutation. As measured by tumor volume TF-targeting CAR1-NK cells suppressed orthotopic PDX growth. Ex vivo tumor weights also demonstrated a substantial difference (55).

In summary, MSLN and TF have shown promise as targets for CAR-NK cell therapy in TNBC. These studies provide valuable insights into potential avenues for developing effective immunotherapies against triple-negative breast cancer. Further research and clinical trials are warranted to explore the translational potential of these findings for TNBC patients.

### 3.4 Pancreatic cancer

PC is the third most common cause of cancer death worldwide, and the five-year survival rate is less than 7%, which is the lowest among all cancers (75–77). Despite many advances, PC patients have an extremely poor prognosis due to late diagnosis, aggressive tumor growth, and resistance to conventional treatments (78). Consequently, there is an urgent need for innovative therapeutics to offer hope to our loved ones (79–81).

Previous research has highlighted stimulator of interferon gene (STING) agonists, tumor-sensing properties, and antitumor efficacy in various cancers such as B16 melanoma, CT26 colon cancer, and 4T1 breast (82–84). However, the exact pathway and direct impact on NK cells have been explored only in a handful of studies. On a parallel note, CAR-NK therapy targeting MSLN has demonstrated strong cytotoxicity in pancreatic cancer cells (85–89).

To examine the synergistic response of both therapies, Da et al. (90) determined the potential use of STING agonist cyclic GMP-AMP (cGAMP) and mesothelin targeting CAR-NK-92 cell therapy in both vitro and *in vivo* model systems. The authors constructed second-generation anti-MSLN CAR-NK-92 cells having anti-MSLNscFv, CD8α as the intracellular domain, 4-1 BB, and CD3ζ as a costimulatory domain. Subsequently, they assessed the stimulatory effect of cGAMP on the pancreatic cancer cell line (AsPc-1) co-cultured with anti-MSLN CAR-NK-92 cells. The results revealed that the STING agonist cGAMP stimulates the antitumor effector capabilities of NK cells in two ways (90).

At first, by directly triggering STING signaling in NK cells treated with cGAMP, observing increased cytotoxicity marked by up-regulation of IFN-β, CD69, CD107a, perforin, granzyme B, and cytokines IFN-γ and TNF-α. This directly induced apoptosis through the intrinsic pathway, as confirmed by the down-regulation of Bcl2 and up-regulation of Bax (91, 92). Secondly, cGAMP indirectly activates NK cells by heightening the sensitivity of pancreatic cancer cells, leading to increased NKG2D ligand expression and chemokine synthesis. Consequently, cGAMP enhances the susceptibility of tumor cells to NK cell cytotoxicity, indirectly activating NK cells.

Next, the authors investigated the combined effects of cGAMP and anti-MSLN CAR-NK-92 cells in an *in vivo* setting. Female NOD-SCID mice, aged 6 to 8 weeks, were subcutaneously injected with AsPC-1 cells. When the tumor volume reached 100 mm^3^, the mice were categorized into different treatment groups, each receiving one among cGAMP, NK-92 cells, cGAMP along with NK-92 cells and anti-MSLN CAR-NK-92 cells in combination with cGAMP (combination group). The mice received NK cell treatment via the tail vein and IL-2 intraperitoneally every 3 days, with cGAMP injected intratumorally three times a week. The results showed that the combination group exhibited a more pronounced inhibitory effect on tumor growth than other groups.

Thus, the findings above indicate that cGAMP may improve pancreatic cancer cells’ susceptibility to NK cell cytotoxicity in addition to directly boosting NK cells’ antitumor activity hence could develop into a unique therapeutic approach for pancreatic tumors in clinical settings.

Similarly, Lee et al. (93) devised allogenic CAR-NK cells targeting folate receptor alpha (FRα, also known as FOLR1), a glycosylphosphatidylinositol (GPI)-anchored membrane protein prevalent in various human cancers (93–96). Simultaneously, they explored death receptor 4 (DR4 or TRAIL receptor-1), situated in the cytoplasmic region, capable of selectively inducing apoptosis in cancer cells through receptor/ligand complex formation (97–101). A previous study identified FOLR1 and DR4 as potential diagnostic markers for pancreatic cancer (PC) patients, showcasing a negative correlation in their expressions (102–104).

Subsequently, four CARs were constructed—NK-GFP, NK-FRα CAR, NK-TRAIL, and NK-Combi. NK-FRα CAR and NK-Combi were subjected to experimentation to assess antitumor efficacy, while NK-GFP and NK-Trail served as negative and positive controls, respectively. The NK-FRα CAR-NK cell featured an antihuman FRα-specific C4 scFv linked to CD27 and CD3ζ signaling domains, preceded by a GFP reporter (105). Additionally, the TRAIL gene was incorporated into NK-Combi, leveraging natural, surface-displayed TRAIL for apoptosis induction even in cancer-resistant cells (106).

The cytotoxic effect of CAR-NK cells was then evaluated against patient-derived pancreatic cancer cell lines (PDC) and human pancreatic cancer cell lines. Notably, NK-Combi exhibited twice the cytotoxicity of NK-FRα CAR and other CAR NK cells at a 5:1 ratio, particularly against PDC lines expressing both FRα and DR4/5 antigens on their cell surface (93). The study concluded that NK-Combi, armed with cytotoxic reagents, demonstrated heightened cancer-selective cytotoxicity. Analysis of CD107a surface expression revealed strong activation in NK-Combi cells, along with increased levels of TNFα, IFNγ, and cytokine production (107). These data suggest that TRAIL-anchoring NK-Combi cells effectively produced cytotoxic effector molecules, resulting in heightened cytotoxicity against tumor cells (107–109).

Further, the biodistribution of NK-FRα CAR cells was examined in female BALB/c nude mice. Results indicated preferential accumulation in FRα-positive tumors, and infiltration tests confirmed specific recognition of FRα-positive PC cells. In an 8-week-old NOD-scid IL2rγnull (NSG) mice xenograft model, NK-Combi exhibited substantial tumor reduction and increased cytotoxicity against cells expressing FRα and DR4/5 antigens, triggering cancer-specific extrinsic apoptosis. Cleaved forms of caspase-3 and caspase-8 were identified at tumor sites, indicating cell death via sequential caspase activation (93). Without IL-2 administration, tumor regrowth occurred after 18 days post-treatment with CAR-NK cells, emphasizing the need for repeated IL-2 injections to enhance NK cell longevity and anticancer activity (110, 111).

In summary, the above data suggests that when reprogrammed to target FRα and express TRAIL as a cytotoxic agent, allogeneic NK cells demonstrate potent cytotoxicity against FRα and DR4/5-double positive tumors both *in vitro* and *in vivo*.

Another potential marker is prostate stem cell antigen (PSCA), which is a surface protein on cells anchored by glycosylphosphatidylinositol, playing a major role in intracellular signaling and the proliferation of tumors (112). To validate this, Teng et al. (113), using immunohistochemistry, observed the expression of PSCA in both pancreatic tumors and neighboring normal tissues. Among the 12 tumor samples obtained from patients, nine (75%) exhibited positive PSCA expression, with seven samples showing strong positivity and two displaying weak positivity (113). In contrast, non-malignant cells in the adjacent tissues demonstrated minimal or negligible PSCA expression. After confirmation of the target, the PSCA CAR was engineered, including, in sequential order, a signal peptide (SP), anti-PSCA single scFv, hinge, transmembrane domain (TM), CD28, and CD3ζ. The anti-PSCA scFv-hinge-CD28-CD3ζ-2A sequence, spanning 1517 base pairs, was inserted into the RRV vector at the NotI/SalI site. To leverage the activating and survival-enhancing effect on NK cells, a soluble form of IL-15 (s15) was integrated into the PSCA CAR construct (114, 115). The codon-optimized s15 was connected to CD3ζ. Another significant modification performed to reduce the toxicity caused by CAR NK therapy was the insertion of a truncated EGFR (tEGFR), serving as a traceable marker and an *in vivo* suicide switch. It was sequentially fused to s15 through the porcine echovirus-1 2A-like self-cleaving peptide (P2A) (116, 117). The fragment containing IL-15-2A-tEGFR was inserted into the RRV at the SalI/MluI site. At last, the retrovirus expressing PSCA CAR_s15_tEGFR was generated by co-transfecting the retroviral transfer plasmid with pRD114-TR, encoding the envelope glycoprotein, into GP2-293T cells. 

Furthermore, to validate the functionality of the engineered PSCA CAR-s15 NK cells (derived from umbilical cord blood) *in vitro*, the expression of PSCA in different cell lines was inspected. Consequently, one PSCA-positive cell line (Capan-1) and one PSCA-negative cell line (PANC-1) were opted for the experiment. Both pancreatic tumor cell lines were incubated with s15 NK cells, PSCA CAR NK cells, and PSCA CAR-s15 NK cells. Authors observed that the stimulation of IFN-γ and the relative cytotoxicity was notably more potent in PSCA CAR-s15 NK cells, highlighting the combined and additive impact of IL-15 along with the PSCA CAR on IFN-γ induction. On the contrary, no inhibition of PANC-1 cell lines was observed. These findings suggest that the concurrent expression of IL-15 and PSCA CAR on NK cells is advantageous and exhibits specificity in inhibiting the growth of PSCA-positive tumors (114, 115).

Further, the therapeutic efficacy of PSCA CAR-s15 NK cells in male and female NOD scid gamma (NSG) mice lacking immune function, aged 8-12 weeks, were implanted with the Capan-1 or MIA PaCa-2 pancreatic cancer cell line expressing a luciferase_ZsGreen gene (designated as Capan-1_luc or MIA PaCa-2_luc) through intraperitoneal (i.p.) injection. Subsequently, a combination of intraperitoneal (i.p.) and intravenous (i.v.) injections were employed, guided by the reasoning that utilizing both routes of administration would target and eliminate tumor cells in both the pancreas and metastatic sites in the liver and lungs. Therefore, twelve administrations of PSCA CAR-s15 NK cells were distributed over three treatment cycles. Each cycle encompassed four doses administered within two weeks, demonstrating greater therapeutic efficacy in impeding the advancement of metastatic cancer compared to the other treatments. The research also suggests that the pre-prepared PSCA CAR-s15 NK cells can endure in the pancreas and effectively target organs affected by metastatic disease, such as the liver and lungs. This leads to prolonged survival in a metastatic pancreatic cancer model.

The evidence subsequently showcases the promise of PSCA CAR-s15 NK cells as a cryopreserved, readily available product supported by both *in vitro* and *in vivo* data (113, 116).

### 3.5 Glioblastoma

Glioblastoma (GBM) is one of the most common and aggressive types of brain cancer. It is one of the deadliest malignant brain tumors and is highly resistant to therapy. Owing to the inherent tumor heterogeneity, active immunometabolic suppression, and antigen escape mechanisms characteristic of GBM cells, these cells tend to evade immunological detection (118).

In the pursuit of identifying more effective immunotherapy targets for GBM, Wang et al. (2021) identified two potential markers: NT5E (5’-Nucleotidase Ecto), responsible for encoding the enzyme CD73 (cluster of differentiation 73), and *B4GALNT1* gene encodes the enzyme beta-1,4-N-acetyl-galactosaminyltransferase 1, that help in the production of glycolipid disialoganglioside (GD2) (118, 119). CD73 performs an essential role in tumor growth with the help of extracellular adenosine production, and GD2, a well-known tumor-associated glycolipid disialoganglioside, is found in malignant glioma and various other cancer types (119, 120). Analyses of the Cancer Genome Atlas (TCGA) database of GBM patients, revealed that GD2 and CD73 jointly have a more pronounced negative impact on patient prognosis compared to GD2 expression alone (118).

The study also revealed a correlation between CD73 expression and NK cell activation, where CD73 expression leads to the suppression of the NK cell-activating receptor NKG2D involving ligands MICA or MICB. The shedding of these ligands MICA and MICB promotes the survival of tumor cells in hypoxic conditions through adenosine (ADO) buildup, impairing NK cell function via purinergic signaling (121). Further investigation into the functional relationship among CD73, GD2, and the presence and activation of NK cells in GBM was carried out using the signature gene set of NK cell (NCR1, NCR3, KLRB1, CD160, and PRF1). The results indicated a negative correlation between NT5E and B4GALNT1, while a positive correlation was observed between MICA and MICB (NKG2D ligand-encoding genes) and individual NK signature genes (NCR1, NCR3, KLRB1, CD160, PRF1) (118).

In the pursuit of concurrently targeting GBM antigens (CD73, GD2, and NKG2DL), the researchers constructed a bicistronic vector expressing two distinct CARs: a GD2.CD28.CD3ζ-CAR and an NKG2D.DAP10.CD3ζ-CAR in NK cells. Subsequently, they further modified the GD2.CD28.CD3ζ-CAR by attaching an anti-CD73 single-chain variable fragment (scFv) through a cleavable, tumor-sensitive linker, creating the CD73 scFv-GD2.CD28.CD3ζ-CAR. Engineered NK-92 cells expressing these constructs exhibited significantly increased killing of GBM43 cells and reduced CD73 enzymatic activity, indicating functional anti-CD73 scFv capable of targeting CD73 and preventing adenosine accumulation. These findings highlight the potential of CD73, GD2, and NKG2DL as combined targets for GBM, forming a foundation for further exploration in NK cell-based GBM immunotherapy (118).

The *in vivo* antitumor effect of CD73.mCAR-pNK cells were subsequently assessed in a subcutaneous xenograft model, introducing patient-derived GBM43 cells into NOD-Rag (-)-gamma chain (-) (NRG) mice. Mice treated with primary NK (pNK) cells exhibited substantially slowed tumor growth compared to the control group, with higher numbers of NK cells found in tumors treated with CD73.mCAR-pNK cells. Immunofluorescence staining revealed locally cleaved anti-CD73 scFv in CD73.mCAR-pNK cell–treated mice near the tumor, indicating an effective reduction of CD73 on cancer cells following engineered NK cell treatment. The combined therapy of chloroquine (CQ) + CD73.mCAR-pNK cells showed a notable suppression in tumor growth, indicating the most significant benefit in the orthotopic model of GBM in NRG mice (118).

In a related context, Zhang et al. (122) explored the potential of ErbB2-targeted CAR NK cells as a glioblastoma treatment using NK-92/5.28.z cells. ErbB2 expression was assessed in 56 primary human glioblastoma tissue samples, revealing its potential as a therapeutic focus. *In vitro*, studies have shown that modified NK-92/5.28.z efficiently and selectively killed ErbB2-positive human GBM cells (122).

The study also evaluated the *in vivo* efficacy of NK-92/5.28.z using NSG mice with subcutaneous LN-319 glioblastoma xenografts. The therapy led to quick tumor regression and statistically significant increases in survival times compared to controls. Subsequently, authors found that NK-92/5.28.z cells effectively suppress the growth of subcutaneous glioblastoma tumors in immune-competent mice. Furthermore, the mice treated with these cells demonstrated an innate, tumor-specific immune response (122).

In a separate investigation, Strecker et al. (123) assessed the functional attributes and immune-modulatory impacts of HER2-targeted anti-HER2 CAR/NK-92 cells and HER2-AAV that stimulates anti-PD-1 expression (123). *In vitro* studies found that anti-PD-1 encodes HER2- AAVs and anti-HER2.CAR/NK-92 cells significantly kill HER2+. The study found a combination of anti-HER2.CAR/NK-92 cell therapy and HER2-AAV anti-PD−1 effectively controlled tumor growth and extended survival in both subcutaneous GL261-HER2 and Tu2449-HER2 glioma models. This approach exhibited a synergistic antitumor impact, indicating its potential as a treatment option. These findings demonstrate the importance of further research into this combined treatment approach. Anti-HER2.CAR/NK-92 cell therapy and HER2-AAV anti-PD−1 effectively control tumor growth and extend survival. They have a synergistic antitumor impact in GL261-HER2 and Tu2449-HER2 glioma models. This approach could be a potential treatment option. The utilization of HER2-AAV in conjunction with anti-HER2.CAR/NK-92 cells as a local therapeutic approach appears to be a potentially innovative strategy for glioma immunotherapy.

### 3.6 Lung cancer

Globally, LC is the second most commonly diagnosed cancer, with an incidence rate of 11.4% and the leading cause of death in 2020 (18%). Lung cancer is classified into non-small cell carcinoma (NSCC) and small cell carcinoma (SCLC), of which NSCC accounts for eighty percent of instances and the latter for the remaining twenty percent (124). Between these two types, non-small cell lung cancer has a poor prognosis and a higher tendency for metastasis (125). Therefore, considering the present scenario of cases that have grown in leaps and bounds in the past centuries, there is an urgent requirement to combat such havoc.

Recently, the B7 homolog three protein (B7-H3, also known as CD276) has emerged as a significant immunoregulatory target for cancer. It has limited expression in normal tissues and shapes the tumor microenvironment (TME) while modulating T cell and natural killer (NK) cell activity, helping the tumor evade the immune system. Due to its overexpression in various tumor types, including lung, breast, and colon cancer, B7-H3 is considered a valuable immunotherapeutic target (126, 127).

In a study by Yang et al. (2020), the researchers recognized the immunotherapeutic potential of B7-H3 in lung cancer (128). They developed a second-generation chimeric antigen receptor (CAR) incorporating 4-1BB and CD3ζ intracellular domains, a CD8 transmembrane region, and an anti-B7-H3 single-chain variable fragment (scFv) construct. The CAR construct was introduced into human NK-92MI cells, a cell line derived from NK-92 cells transfected with human interleukin (IL-2).

To evaluate the cytotoxic effect of CAR construction on A549, NCI-H23, and HCC827 lung cancer cell lines, the authors employed a Calcein-AM test. Results indicated a significant increase in cytotoxicity for CAR-NK-92MI cells in comparison to unmodified NK-92MI cells across different E/T ratios. However, no notable difference was observed at an effector to target (E/T) ratio of 1:1 for MDA-MB-231. Additionally, neither NK-92 derivatives could lyse B7-H3-negative Daudi cells. Further investigations revealed that unmodified NK-92MI cells exhibited moderate killing of HCC827 and MDA-MB-231 cell lines at high E/T ratios, whereas CAR-NK-92MI cells displayed significantly enhanced cytotoxicity. These findings demonstrated the cytotoxicity of B7-H3 CAR-NK-92MI against B7-H3 positive tumor cells. In subsequent flow cytometry experiments, increased levels of granzyme B and perforin were observed at an E/T ratio of 1:1 for 24 hours. Interestingly, CD107a levels in A549 were lower than in NCI-H23, despite higher B7-H3 expression in A549. This suggested that B7-H3 density beyond a certain threshold does not determine cytolytic granule polarization and degranulation. Overall, the study emphasized the crucial role of degranulation activation in the cytotoxicity of CAR-NK-92MI cells (128).

To validate the efficacy in a mouse model, tumor-bearing mice were treated with CAR-NK-92MI cells, unmodified NK-92MI cells, or PBS. The CAR-NK-92MI group exhibited a significant reduction in tumor volume compared to the unmodified NK-92MI group and the PBS group, indicating the potential of CAR-NK-92MI cells in suppressing tumor progression both *in vitro* and *in vivo*. In summary, the study highlights the promise of B7-H3 as an immunotherapeutic target in lung cancer. It demonstrates the effectiveness of B7-H3 CAR-NK-92MI cells in preclinical models, supporting their potential as a therapeutic strategy for combating lung cancer.

Exploring another potential target, Fang et al. (129) investigated fibroblast activation protein (FAP) as a therapeutic target for NSCLC (129, 130). FAP, expressed in 90% of the stroma of epithelial malignant tumors, emerged as a promising target for cancer imaging and treatment in previous studies (130–134). To explore the impact of intervention in NSCLC, the authors employed a pLenti-EF1α-anti-hFAP-CAR lentiviral expression vector to transfect NK-92 cells, resulting in the generation of FAP-CAR-NK-92 cells. The constructed anti-FAP CAR included signaling domains (4-1BB and CD3ζ), an anti-FAP targeting moiety (scFv), CD8α hinge, and CD8 transmembrane domain (TM) (135).

FAP-CAR-NK-92 cells demonstrated heightened cytotoxicity against NSCLC cells expressing FAP. Flow cytometry and an LDH release assay conducted *in vitro* confirmed the efficacy of FAP-CAR-NK-92 cells against FAP-expressing cells. FAP expression rates in A549-hFAP cells, H226-hFAP cells, and CAF-hFAP cells were notably higher (80.62%, 86.45%, and 75.63%, respectively) compared to A549 cells, H226 cells, and CAF cells. The LDH release assay revealed that the killing efficiency of hFAP-CAR-NK-92 cells surpassed that of NK-92 cells. Additionally, the A549-hFAP, H226-hFAP, and CAF-hFAP groups exhibited significantly greater CD107a expression in hFAP-CAR-NK-92 cells, while the K562 group showed no changes compared to NK-92 cells. Pathway analysis using an IHC assay unveiled higher GSDME expression in lung adenocarcinoma tissues in comparison to lung squamous cell carcinoma tissues. Moreover, significantly increased GSDME and Caspase 3 cleavage levels were observed in A549-hFAP, H226-hFAP, and CAF-hFAP cells, indicating that hFAP-CAR-NK-92 cells induced pyroptosis through caspase/GSDME activation. The findings collectively underscored that hFAP-CAR-NK-92 cells induce pyroptosis through caspase/GSDME activation (85, 129).

*In vivo* safety and effectiveness of hFAP-CAR-NK-92 cell treatment were assessed using a tumorigenesis model in NOD-SCID mice. A caudal vein injection of irradiated NK-92 cells in sterile PBS (NK-92 group) and irradiated parental NK-92 cells in sterile PBS (hFAP-CAR-NK-92 group) was administered to the animals in each group (136) on days 12, 19, and 26 after the tumor volume was measured. The mice were sacrificed on day 30, and the tumors were removed for examination, which demonstrated approximate 50% reduction in tumor growth by hFAP-CAR-NK-92 cells than by NK-92 cells.

In a similar study, Chambers et al. (137) engineered NK cells to target CD73, an enzyme-producing adenosine abundantly expressed in NSCLC (137–139). To specifically target CD73, a chimeric antigen receptor (CAR) NK cell was developed, incorporating a CD73 single-chain variable fragment (scFv) with functional and neutralizing properties. The intracellular and transmembrane regions were derived from FC (gamma)RIIIa (CD16) (140–143).

*In vitro* studies comparing CD73 CAR NK cells to control primary NK cells demonstrated significantly heightened cytotoxicity against A549 cells. CAR NK cells produced through either the pcDNA3.1(+) or pLV plasmids generating CD73.mRNACAR and CD73.CAR respectively displayed increased killing efficiency. These CAR NK cells also exhibited elevated CD107a expression and cytokine release, particularly IFN-γ. Moreover, the authors noticed that CD73.mRNACAR-NK cells did not selectively destroy normal HUVEC cells. In general, primary NK cells displayed minimal cytotoxicity against healthy endothelial cells, possibly attributed to factors such as the protective role of ATP and the expression of HLA-E. This suggested that the recognition of CD73 might not be the exclusive mechanism for eliminating target cells by CD73-targeting NK cells. CD73-CAR-NK cells effectively reduced adenosine production in lower baseline levels in A549 cells cultured alone (137).

In an *in vivo* setting using a subcutaneous xenograft model, the anti-CD73 engineered NK cells demonstrated remarkable effectiveness against tumors. When lung adenocarcinoma CD73+ A549 cells were implanted into NRG mice, CD73.CAR-NK cells exhibited a significantly more robust antitumor response than control mice receiving PBS or non-engineered NK cells. This resulted in reduced growth of CD73+ tumors, accompanied by higher levels of granzyme B. Furthermore, CD73-CAR-NK cells led to a prolonged arrest of tumor growth, persisting for nearly 50 days post-tumor implantation, unlike the other treatment groups. Importantly, there was no notable decrease in the body weight of mice across any of the groups during or following the treatment period. In conclusion, the CD73-CAR NK cell therapy approach holds the potential to improve the survival of patients with NSCLC and other CD73+ solid tumors (137).

In another study, researchers developed third-generation c-Met-CAR-NK cells utilizing c-Met-scFv, incorporating Anti-c-Met scFv, the transmembrane domain of NKG2D, and cytoplasmic signaling domains of CD137, 2B4, DAP10, and CD3ζ (41). c-Met, a well-known proto-oncogene encoded by human chromosome 7 (7q21–q31), plays a crucial role in the growth and development of various solid tumors (144–147). The construction of the pCDH-c-Met-CAR-GFP plasmid involved inserting the DNA fragment into the lentiviral vector pRRL-GFP (144). The resulting plasmids (pCDH-c-Met-CAR-GFP, psPAX2, and pMD2G) were transduced into 293T cells to generate c-Met-CAR lentivirus. Western blot analysis confirmed the successful exogenous expression of CD3ζ. Additionally, the expression of chemokines (CCL4, CCL5, IL-15, IL-6) and activating receptors (KLRC2, KLRC3, NCR1, NCR3) was upregulated, while inhibitory receptors (TIGIT and CD96) were downregulated in the CCN4 group, highlighting the complexity of the signaling pathway. The research demonstrated that the CCN4 construct containing DAP10 exhibited superior cytotoxicity compared to other CAR NK cells. The LDH assay revealed that CCN4 achieved a greater specific lysis percentage than NK92 and a 50% increase compared to CCN1, showcasing a more potent cytotoxic effect on the lung adenocarcinoma (LUAD) cell line with minimal impact on the control (HBE cell line). These results suggest the potential efficacy of CCN4 in a mouse model.

To validate the *in vivo* effect of c-Met CAR NK cells, six to eight-week-old nude mice were subcutaneously administered with H1299 cells to grow tumors up to 50 cm^3^. Treatment with CCN4 yielded the most promising results, with an approximately 69% decrease in tumor weight compared to NK92 cells and a 33% decrease compared to CCN1. Immunohistochemistry (IHC) analysis supported these findings, revealing increased CD56, perforin, and granzyme B protein expression, indicating CCN4-induced tumor necrosis and upregulated NK cell infiltration. Collectively, these findings suggest that c-Met CAR NK cells hold the potential to effectively eliminate tumors and extend the survival of tumor-bearing mice (41).

In a similar approach targeting small cell lung cancer (SCLC), researchers investigated the therapeutic potential of CAR-engineered NK-92 cells directed against delta-like ligand 3 (DLL3). DLL3 is expressed more in SCLC patients than normal adult tissues (148–150). An immunohistochemistry (IHC) study on 50 SCLC samples revealed that 76% had DLL3 protein, with 54% showing moderate to high expression, suggesting that DLL3 could be an effective target for treatment. For this study, DLL3- or CD19-CAR molecules were generated, consisting of the CD8 leader, scFv of DLL3 or CD19, a CD8 hinge, an NKG2D transmembrane region, and the costimulatory domains of 2B4 and CD3ζ. NK-92 cells were transduced with the DLL3- or CD19-specific CAR vector. Results demonstrated that DLL3-CAR NK-92 cells exhibited higher cytotoxicity than CD19-CAR NK-92 or parental NK-92 cells when co-cultured with H209 GL and H446 GL cell lines. Cytokine-secreting tests revealed significant secretion of IFNγ, granzyme B, GM-CSF, and perforin associated with CAR-induced lysis.

*In vivo* experiments in 6 to 8 weeks old NSG mice utilized intrapulmonary SCLC and subcutaneous xenograft models. DLL3- or CD19-CAR NK-92 cells were administered intravenously to mice with tumor volumes ranging from 50 to 100 mm³ every seven days. The DLL3-CAR NK-92 group exhibited prolonged survival compared to the control and CD19-CAR NK-92 groups. No overt harm was observed in the vital organs of mice receiving CAR NK-92 cell treatment. DLL3-CAR NK-92 cells extended the survival period of tumor-bearing mice and efficiently destroyed cell line-derived pulmonary SCLC cells *in vivo*, suggesting potential for tumor regression. Moreover, high IFN-γ release in mice serum and the absence of IL-6, a leading cause of cytokine release syndrome (CRS), in the subcutaneous mouse model indicated that DLL-3 CAR NK-92 treatment could effectively eliminate tumors with minimal side effects (148).

In a related study by Xu et al. (151), researchers explored the synergistic effects of CAR NK cell therapy combined with adjuvant photothermal therapy (PTT) using penta-modal imaging. Mice were subjected to treatment involving upconversion nanoparticles (UCNPs) and IR-1048 dye incorporated into the lipid-aptamer nanostructure (UCILA), Laser, and B7-H3-specific CAR-NK-92MI cells. The results indicated inhibited tumor growth with no relapse or metastasis in other organs, showcasing promising outcomes for the combination therapy (151).

### 3.7 Liver cancer

Liver cancer stands as the fourth major cause of cancer-related fatalities and ranks sixth among the most common cancers globally (152, 153). The majority of primary types of liver cancers are hepatocellular carcinoma (HCC), intrahepatic cholangiocarcinoma (iCCA), fibrolamellar carcinoma, and hepatoblastoma (154, 155). However, HCC comprises 70% to 90% of all types of primary liver cancers (156). Therefore, there is an urgent need for novel approaches for treating advanced HCC patients (157).

Conventional treatments, including CAR-T cell therapy, have limitations due to complications like graft-versus-host disease (GVHD) (158–160). As an alternative, CAR-NK therapy has arisen as a viable immunotherapeutic strategy for HCC (157). The oncofetal glycoprotein Glypican 3 (GPC3) is highly expressed in HCC, making it a potential immunotherapy target (161, 162).

Thus, to investigate the immunotherapeutic approach for the treatment of HCC, Yu Min et al. (157) developed GPC3-specific CAR-NK cells. In this regard, authors developed GPC3-specific CAR-NK cells using a pRRLSIN lentiviral vector backbone. The CAR construct included a CD8 alpha signal peptide, a humanized single-chain variable fragment (scFv) named hu9F2 targeting GPC3, a CD8 alpha hinge region, CD28 transmembrane region, CD28 co-stimulatory intracellular domain, and CD3ζ intracellular domain. Expression of GPC3-specific CAR in NK-92/9.28.z was confirmed with the help of flow cytometry analysis. The engineered NK-92/9.28.z cells demonstrated cytotoxicity effects against GPC3-positive HCC cell lines, including HepG2, Huh-7, Hep3B, and PLC/PRF/5, while sparing GPC3-negative cells and showing no impact on GPC3- SK-HEP-1 and SMMC-7721 cells (157).

Further, they investigated the anti-tumor effect of NK-92/9.28.z cells *in vivo* using NOD/SCID mice with subcutaneous SK-HEP-1 and SK-HEP-1/GPC3 xenografts.The introduction of NK-92/9.28.z cells significantly suppressed the growth of the SK-HEP-1/GPC3 xenografts but had no impact on the growth of the SK-HEP-1 tumors. They also explored the capability of NK-92/9.28.z cells to target existing GPC3+ tumors. NK-92/9.28.z cells showed a significant accumulation within the SK-HEP-1/GPC3 xenografts, whereas only a small number of NK cells were found in the tumors in mice injected with parental NK-92 cells. Following intravenous administration of NK cells, IHC test results verified the accumulation of NK-92/9.28.z cells within the remaining SK-HEP-1/GPC3 tumors. In contrast, tumors subjected to NK-92 cell treatment displayed a diminished presence of NK-92 cells, and there was no identifiable staining in the tumor sections from mice treated with PBS. SK-HEP-1/GPC3 tumors extracted from NK-92/9.28.z-treated mice showed a major decrease in proliferation as evaluated by Ki67 staining and an increase in apoptosis as indicated by cleaved caspase-3 staining. Furthermore, after receiving the above therapies, H&E staining was used to analyze organs (heart, liver, lung, kidney, and pancreas) from SK-HEP-1/GPC3-bearing mice, and no evident tissue damage was found in any group (157).

*In vivo* experiments using NOD/SCID mice with SK-HEP-1/GPC3 xenografts demonstrated a substantial suppression of tumor growth upon treatment with NK-92/9.28.z cells. The CAR-NK cells also showed tumor-specific accumulation and induced apoptosis in GPC3-positive tumors without causing evident tissue damage in other organs.

Further validation was performed in orthotopic xenograft models with endogenous GPC3 expression, showing reduced tumor development. Additionally, irradiated NK-92/9.28.z cells exhibited significant tumor-targeting capabilities against PLC/PRF/5 xenografts, even after radiation exposure, suggesting their potential for safe utilization *in vivo*. The study highlighted the improved cytotoxicity of CAR-NK cells against HCC cells while minimizing toxicity to non-tumor cells, making them a promising therapeutic option for advanced HCC patients. Introducing CAR (9.28.z) into NK cells enhanced their cytotoxicity against cancerous cells while demonstrating minimal toxicity towards non-transformed cells (157).

In summary, GPC3-specific CAR-NK cells show great potential for targeted immunotherapy in HCC, providing a novel and effective approach for treating this challenging cancer like liver cancer.

### 3.8 Colorectal cancer

CRC ranks second in terms of cancer-related deaths globally and is the third most frequent type of cancer, with 1.1 million new cases reported in the year 2020 (2).

To assess the anticancer potential of CAR-NK in humanized CRC mouse models (163), Zhang et al. (163) engineered second-generation EpCAM-specific CAR-NK-92 cells. Notably, over 97% of colorectal cancer patients exhibit elevated expression of epithelial cellular adhesion molecules (EpCAM), contributing to cell proliferation and metastasis (164). The authors designed an EpCAM-specific scFv fused to a CD8 hinge, transmembrane domains of CAR-NK-92, and intracellular signaling domains of 4-1BB and CD3ζ in a lentiviral vector system containing green fluorescent protein (GFP) encoding sequences.

Two cell lines overexpressing human EpCAM, namely 293T-EpCAM and FHC-EpCAM, were generated using human embryonic kidney epithelial cell line 293T and human colonic epithelial cell line FHC. Cytokine release experiments demonstrated that CAR-NK-92 cells could selectively identify and activate EpCAM-positive cells, leading to higher cytokine levels (IFN-γ, perforin, and granzyme B) compared to Ctrl-NK-92 cells. LDH release assays revealed that CAR-NK-92 cells exhibited enhanced killing ability against EpCAM-positive colorectal cancer cells at various E:T ratios of 40:1, 20:1, 10:1, 5:1, and 1:1. At the same time, no significant difference was observed in the cytotoxicity towards EpCAM-negative Ctrl NK-92 cells.

To evaluate *in vivo* efficacy, a subcutaneous xenograft model using HCT-8-Luc cells in female NOD/SCID mice was employed. Treatment with Ctrl-NK-92 cells, CAR-NK-92 cells, regorafenib, or the combination of CAR-NK-92 cells and regorafenib remarkably reduced the growth rate of HCT-8-Luc tumors compared to the untreated group. EpCAM-specific CAR-NK-92 cells showed substantial inhibition of tumor growth compared to Control-NK-92 cells. Importantly, the combination of CAR-NK-92 cells and regorafenib dramatically slowed tumor growth, nearly eliminating tumors in mice. Regorafenib, an approved drug for metastatic colorectal cancer and stromal tumors, inhibits receptor tyrosine kinases associated with angiogenesis and tumor microenvironment maintenance. In summary, the study demonstrates the selective killing effect of EpCAM-specific CAR-NK-92 cells on colorectal cancer cells *in vitro* and highlights the potent combination therapy of CAR-NK-92 cells and regorafenib in suppressing tumor growth in a humanized CRC mouse model.

In a similar study performed by Shiozawa et al. (165), the NK cell line NK-92MI was utilized to target tumors expressing carcinoembryonic antigen (CEA) (165). NK-92MI is an interleukin-2-independent (IL-2-independent) cell line, generated by introducing human IL-2 cDNA into NK-92, sharing characteristics with NK-92 and activated NK cells (166). Previous studies have demonstrated the potent cytotoxicity of both NK-92 and NK-92MI cells against human melanoma cells *in vitro* and *in vivo* (167, 168). Carcinoembryonic antigen (CEA) overexpression has been associated with the progression of human cancer, influencing processes such as anoikis suppression, apoptosis, and cell differentiation (169–171).

The authors successfully transduced an anti-CEA-specific single-chain variable antibody fragment (scFv) into NK-92MI cells in this study. The anti-CEA-specific CAR was constructed by assembling the pGEM-1 plasmid with the cDNAs of the variable heavy-chain (VH) and light-chain (VL) domains of the humanized monoclonal anti-CEA antibody, the CD8α hinge region, and the transmembrane and intracellular domains of CD3ζ.Their findings suggest that anti-CEA-CAR modified NK-92MI cells hold potential for clinical therapy in treating advanced colorectal cancer.

A xenogeneic model using nine-week-old female SCID mice was further employed to validate the *in vivo* therapeutic activity of anti-CEA-CAR NK-92MI cells. Treatment with NK-92MI or NaB (sodium butyrate) alone did not confer therapeutic benefits. However, anti-CEA-CAR NK-92MI cell treatment substantially inhibited tumor growth, irrespective of NaB use. Tumors treated with anti-CEA-CAR NK-92MI cells were notably smaller than those in the control groups. Notably, combining NaB with anti-CEA-CAR NK-92MI cells resulted in significantly reduced tumor sizes, a finding of significance compared to the control group.

### 3.9 Renal cell carcinoma

According to GLOBOCAN 2020 data, kidney cancer accounted for 2.2% of cancer incidences and 1.8% of mortality. Renal cell carcinoma (RCC), a subtype of kidney cancer, is complicated due to its resistance to chemotherapy and radiotherapy. Moreover, patients undergoing radical resection often encounter issues of metastasis and recurrence (172–174).

In the pursuit of innovative immunotherapeutic strategies for RCC patients, Zhang et al. (175) explored the combined efficacy of third-generation EGFR-specific CAR-NK-92 cells transduced by lentiviral and the chemotherapeutic drug cabozantinib in a mouse model of human RCC (175). Cabozantinib, an FDA-approved tyrosine kinase inhibitor (TKI), exhibits antitumor activity while altering the TME by reducing the function of myeloid-derived suppressor cells (MDSCs) and regulatory T cells (Tregs) for treating advanced RCC (176–178).

After confirming CAR-NK expression, researchers went for *in vitro* analysis using human renal cancer cell lines 786-O, ACHN, and EGFR-negative colorectal cancer cell lines as control (179, 180). *In vitro* studies demonstrated that EGFR-positive kidney carcinoma cells could be selectively identified and killed by CAR-NK-92 cells. Cytokine release tests indicated a significant increase in IFN-γ, perforin, and granzyme B, in CAR-NK-92 cells compared to Ctrl-NK-92 cells. The research findings indicated that cabozantinib modified the expression of cell-surface markers, rendering renal cancer cells more prone to targeted attack by CAR-NK-92 cells, allowing for their precise identification and elimination.

For *in vivo* validation, a subcutaneous xenograft model using 786-O cells and human renal cancer ACHN cells was established in NOD/SCID mice. The mice received subcutaneous administration of cell lines for tumor growth, followed by cabozantinib therapy five times a week. The Ctrl-NK-92, CAR-NK-92, and CAR-NK-92 + cabozantinib groups received treatment once a week for six times starting one day after the initiation of cabozantinib therapy. All animals received 2000 IU recombinant human IL-2 (rhIL-2) once every other day beginning on the day after NK-92 cell injection. The study’s findings imply that CAR-NK-92, in combination with cabozantinib, can enhance the susceptibility of CAR-NK-92 cell-mediated killing, potentially offering a promising therapeutic approach for RCC patients (175).

Overall, the above study suggests the potential of combining targeted EGFR NK-92 cells with small-molecule inhibitors, such as cabozantinib, to enhance treatment outcomes in kidney cancer, offering a promising therapeutic approach for RCC patients.

## 4 Overview and future facets

Despite the promise of CAR-T cells in treating hematological cancers, their efficacy in addressing solid tumor cells is limited. Additionally, CAR-T immunotherapy’s reliance on apheresis and the growth of patient-derived autologous immune cells introduces delays and challenges, especially when donor T cells are scarce.

Therefore, we propose CAR-NK cells as a more advantageous option than CAR-T cells as they are more cost-effective with improved antitumor activity and significant potential for allogenic off-the-shelf manufacture for cancer therapy. Our comprehensive investigation also indicates that CAR-NK cells can effectively target and regress non-hematological malignancies, showcasing enhanced antitumor efficacy with minimal side effects. The presented information implies a great deal of therapeutic promise for treating tumors using genetically modified NK cells to maximize their potential. Notably, NK cells exhibit low graft versus host disease (GvHD) potential and rarely induce significant toxicities due to limited persistence in the body, lack of T-cell receptors, decreased CRS especially IL-6, and safety of allogenic NK cells, making them a promising platform for CAR engineering. Also, the adoptive transfer of allogenic NK cells into patients further underscores the diversity of NK cells for various applications (181).

In details, we have underlined the importance of CAR-NK cells in recognizing antigens specific to solid malignancies and highlighted preclinical investigations identifying potential target antigens for CAR-NK cell treatment. A few preclinical glioblastoma investigations identified GD2 and CD73 as possible target antigens for CAR-NK cell treatment. Additionally, mesothelin has emerged as a potential target tumor antigen for ovarian, pancreatic, breast, and gastric cancers. The estimated tumor volumes in the MSLN-CAR NK group stayed largely steady and small. The group showed significantly lower average tumor weights than NC, with 0.23 g for gastric cancer and 0.12 g for ovarian cancer. Moreover, combining cGAMP with anti-MSLN CAR-NK-92 cells could enhance pancreatic cancer cells’ cytotoxicity and anti-tumor activity, potentially forming a unique clinical treatment approach. Therapeutic targets for colorectal cancer have been identified, including CEA and EpCAM. Research on several antigens, including c-MET, DLL3, FAP, and B7-H3 (CD276), has been conducted concerning lung cancer treatment. The results demonstrated that caspase/GSDME activation is the mechanism via which hFAP-CAR-NK-92 cells cause pyroptosis in NSCLC cells. Moreover, according to our thorough analysis, patients with NSCLC and other CD73+ solid tumors may have a higher chance of survival when using the CD73-CAR NK cell treatment strategy. Targets for CAR-NK cell treatment in pancreatic cancer includes cGAMP, DR4, and FOLR1. Additionally, CAR-NK cell treatment has been found effective in targeting EGFR and TF in renal cell carcinoma and breast cancer cases, respectively. Targeted immunotherapy in hepatocellular carcinoma holds considerable potential, particularly with GPC3-specific CAR-NK cells.

We have highlighted how crucial it is for CAR-NK cells to overcome some difficulties and their ability to recognize antigens specific to tumors in treating solid malignancies. The encouraging results in preclinical models imply that this approach may have translational implications for treating human cancer. However, it is important to recognize several obstacles to overcome when moving from preclinical research to clinical applications, such as tumor heterogeneity and safety issues like neurological toxicity, immunosuppression, and off-target effects which can damage healthy tissues due to cross-reactivity between the CAR-NK cell receptor and antigens present on normal cells.

Other challenges include the potential incomplete targeting of all tumor cell populations, leading to incomplete cancer regression and the risk of relapse. The short duration of CAR-NK cell activity may necessitate repeated administrations, potentially increasing the risk of adverse events. Lengthy regulatory processes can further hinder the translation of preclinical findings into timely clinical applications.

Genetically modified natural killer cells’ anticancer efficacy in non-hematological solid tumors shows excellent promise as a novel and perhaps successful therapeutic strategy. More investigation and clinical trials are necessary to validate and improve these results and get closer to creating secure and effective treatments for patients with solid tumors. Addressing the challenges requires ongoing research and innovation to enhance the effectiveness and specificity of CAR-NK cells.

We hope the data and knowledge presented in this study will inspire future researchers, clinicians, and pharma companies to develop viable, tailored-made, off-the-shelf NK cells for multiple cancer patients.

## Author contributions

CPD: Investigation, Data curation, Methodology, Visualization, Writing – original draft. DS: Data curation, Investigation, Methodology, Visualization, Writing – original draft. PD: Data curation, Investigation, Methodology, Visualization, Writing – original draft. RY: Data curation, Investigation, Methodology, Writing – original draft. DC: Data curation, Investigation, Methodology, Writing – original draft. BPS: Data curation, Investigation, Methodology, Writing – original draft. PS: Data curation, Investigation, Methodology, Writing – original draft. VU: Data curation, Investigation, Supervision, Writing – review & editing. MJ: Supervision, Writing – review & editing, Conceptualization, Funding acquisition, Project administration, Resources. AJ: Investigation, Project administration, Resources, Supervision, Writing – review & editing, Conceptualization.
